# Transcriptome analysis revealed the drought-responsive genes in Tibetan hulless barley

**DOI:** 10.1186/s12864-016-2685-3

**Published:** 2016-05-20

**Authors:** Xingquan Zeng, Lijun Bai, Zexiu Wei, Hongjun Yuan, Yulin Wang, Qijun Xu, Yawei Tang, Tashi Nyima

**Affiliations:** Tibet Academy of Agricultural and Animal Husbandry Sciences, Lhasa, 850002 China; Barley Improvement and Yak Breeding Key Laboratory of Tibet Autonomous Region, Lhasa, 850002 China; Chengdu Life Baseline Technology Co., LTD, Chengdu, 610041 China

**Keywords:** *Hordeum vuglare*, DGE, Transcriptome sequencing, Drought-responsive genes, ABA, Tetrapyrrole

## Abstract

**Background:**

Hulless barley, also called naked barley, is an important cereal crop worldwide, serving as a healthy food both for human consumption and animal feed. Nevertheless, it often suffered from drought stress during its growth and development, resulting in a drastic reduction in barley yields. Therefore, study on molecular mechanism of hulless barley drought-tolerance is very important for increasing barley production. To investigate molecular mechanism of barley drought-resistance, this study examined co-regulated mRNAs that show a change in expression pattern under early well water, later water deficit and finally water recovery treatments, and to identify mRNAs specific to water limiting conditions.

**Results:**

Total of 853 differentially expressed genes (DEGs) were detected and categorized into nine clusters, in which VI and VIII were apparently up-regulated under low relative soil moisture content (RSMC) level. The majority of genes in these two clusters was relevant to abiotic stress responses in abscisic acid (ABA) dependent and independent signaling pathway, including *NCED*, *PYR/PYL/RCAR*, *SnRK2*, *ABF*, *MYB/MYC*, *AP2/ERF* family, *LEA* and *DHN*. In contrast, genes within clusters II and IV were generally down-regulated under water stress; cluster IX genes were up-regulated during water recovery response to both low and high RSMC levels. Genes in implicated in tetrapyrrole binding, photosystem and photosynthetic membrane were the most affected in cluster IX.

**Conclusion:**

Taken together, our findings indicate that the responses of hulless barley to drought stress shows differences in the pathways and genes activated. Furthermore, all these genes displayed different sensitivities to soil water deficit and might be profitable for future drought tolerance improvement in barley and other crops.

**Electronic supplementary material:**

The online version of this article (doi:10.1186/s12864-016-2685-3) contains supplementary material, which is available to authorized users.

## Background

Hulless barley (*Hordeum vuglare* L. var. *nudum* Hook. f.) is one of the most important crops in China, especially in Tibet Plateau, for over half of the total food production. With caryopses that thresh free from the pales, hulless (naked) barley provides an attractive advantage for the human consumption [1]. It is cultivated in the valleys and in the higher land on Tibet. Drought-induced water deficit greatly affects crop growth and development, and endangers crop agronomic yields. Crops have also formed various molecular and physiological changes to prevent water deficit. Drought tolerance crops maintain turgor and continue metabolism in cells even at low water potential, mainly by protoplasmic tolerance, synthesis of osmolytes or/and compatible solutes [2]. Signal transduction molecules play important roles in this process by mediating the transmission of the stress signals via complex signal transduction pathways. Numerous fundamental molecular aspects of tetrapyrroles and abscisic acid (ABA) are available [3–5]. Tetrapyrroles are the active cores of some compounds with crucial biochemical roles in living systems, such as chlorophyll, heme, siroheme and phytochromobilin [6]. Heme biosynthesis is transcriptionally responsive to reactive oxygen species (ROS)-mediated stress signaling in *Arabidopsis* [7]. ABA is a vital stress-responsive phytohormone sensitive to these cellular changes, particularly to the loss of turgor [8]. The ABA signal transduction pathway comprises the ABA-bound pyrabactin resistance/regulatory component of ABA receptor (*PYR/RCAR*) proteins [9], type 2C protein phosphatases (*PP2C*) [10], and SNF1-related kinases (*SnRK2*) [11], *NCED* [12], *ABF* [13], *MYB/MYC* [14], *AP2/ERF* family [15], *LEA* [16] and *DHN* [17]. In *Arabidopsis*, the molecular drought response mechanism can be divided into ABA-dependent and ABA-independent pathways [18]. In soybean, ABA treatment influences the expression of drought response genes [19].

Breeding for drought tolerance is particularly challenging because of the genetic complexity of this trait. On the one hand, hulless barley has a complex diploid genome (HH), with the genome size of 5000 Mb, larger than that of human [20]. On the other hand, drought tolerance has been well documented to result from cooperative interactions among multiple morphological, physiological, and biochemical characters. Different genotypes may have diverse responses to drought stress [18, 21, 22]. Therefore, efficient improvement requires an indepth understanding of the gene expression regulation mechanisms in response to drought stress.

Many researchers have analyzed Tibetan hulless barley, and many genes associated with drought stress responses in plants are known. Honsdorf et al. [23] has detected the drought tolerance QTL in wild barley, while Chen et al. [24] has analyzed grain development and nutrient storage in Tibetan hulless barley. However, little has been known about display the changes of gene expression in Tibetan hulless barley during the whole drought response process. In the study, we used transcriptome-seq to identify differentially expressed genes in response to drought stress in leaf tissue of a Tibetan hulless barley drought-resistibility cultivar, which were grown in different levels of relative soil moisture content (RSMC) and water recovery. This analysis serves as a reference for future studies on Tibetan hulless barley response to various stresses, such as to drought, cold and salt.

## Results

### Analysis of transcriptome-seq data

In the present study, cDNA libraries were constructed from leaves harvested at eight days after drought stress and during rehydration with three biological replicates, and then sequenced using the Illumina HiSeq™ 2000 platform. After cleaning and checking the read quality, we obtained almost 53.99 billion 200 bp paired-end clean reads. Among the clean reads, 100 % had quality scores at the Cycle Q20 level (a base quality greater than 20 and an error probability of 0.01). The statistics of sequencing sample and data were in Table 1.

**Table 1:** Statistics of transcriptome sequencing data in different samples

Genotype	Samples	Raw Reads (M)	Clean Reads (M)	Q20 (%)	GC (%)
Himalaya 10	A1	75.61	67.79	96.64	55.83
Himalaya 10	B2	75.11	67.95	96.82	54.84
Himalaya 10	C3	75.84	64.22	95.22	55.57
Himalaya 10	D4	75.15	66.79	96.74	55.45
Himalaya 10	E5	73.23	65.40	96.69	56.21
Himalaya 10	F6	75.41	69.19	97.53	54.47
Himalaya 10	G7	74.46	68.07	97.49	55.49
Himalaya 10	H8	73.41	66.20	97.37	56.19
Himalaya 10	I9	69.88	64.24	97.37	56.63

The clean reads of each sample (A1-I9) were mapped to the full gene set of Tibetan hulless barley. The 9 transcriptome data could map to 28,077 genes, which covered 71.6 % of the whole gene-set.

### DEGs analysis and validation of hulless barley sequencing data

Samples A1, D2, C3, D4, E5 and F6 were divided into two groups: one includes A1, B2 and C3, which is under water-sufficient conditions; the other consists of D4, E5 and F6, which is under withholding water conditions. Then, the comparison analysis between the two groups was performed use Noiseq method, and 313 DEGs were finally obtained. The pairwise comparison was also executed among samples F6, G7, H8 and I9. We filtered genes whose RPKM value are below five in all four samples and removed genes that the frequency of occurrence is less than three in pairwise comparison, and then we got 632 DEGs.

**Fig. 1:**
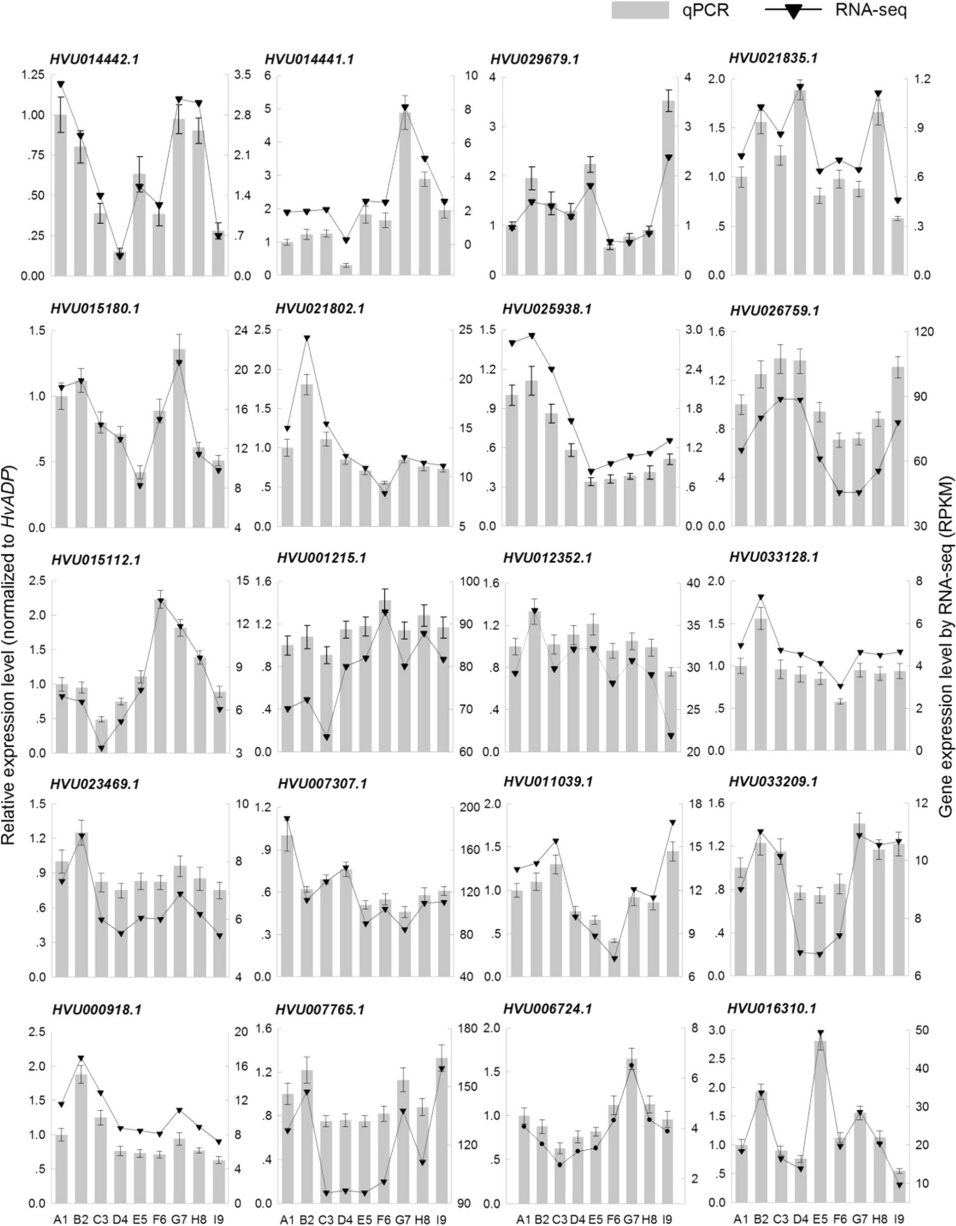
Expression of 20 differentially expressed genes in response to drought stress treatment. Vertical bar charts with simple error bars (left y-axis) represent quantitation of 20 genes transcripts in nine samples, using qPCR. Values are means ± SE (*n* = 3). Line and scatter plot (right y-axis) represents transcript abundance (RPKM) of nine samples for each gene detected by RNA-seq. The A1-F6 indicates the relative soil moisture content (RSMC) of 33.4, 27.5, 21.1, 15.5, 9.8 and 4.8 %, respectively, and G7-I9 indicates the 2 h, 4 h and 8 h after water recovery from 4.8 % to 33.4 %, respectively

Among these DEGs, genes with distinctly changed expression profiling were confirmed using qPCR (Fig. 1). The results of this experiment were basically consistent with RNA-seq data.

### Expression patterns and cluster analysis DEGs

**Fig. 2:**
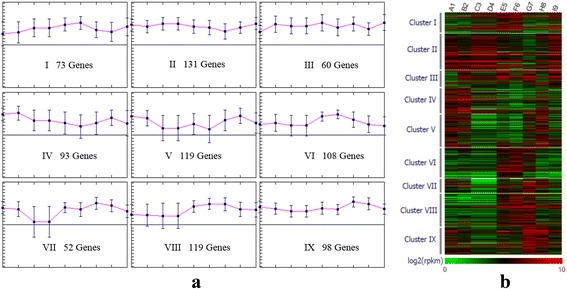
Gene expression pattern for 853 potential drought-resistant related genes clustering into nine clusters. **a** The horizontal axis shows the nine samples A1, B2, C3, D4, E5, F6, G7, H8, I9, while the vertical axis shows the mean value of log-transformed RPKM value for genes in clusters. Error bars were presented for each sample in each cluster. **b** Gene expression pattern for all 853 DEGs. The columns show the nine samples A1, B2, C3, D4, E5, F6, G7, H8, I9, while the rows show the log-transformed RPKM values

**Fig. 3:**
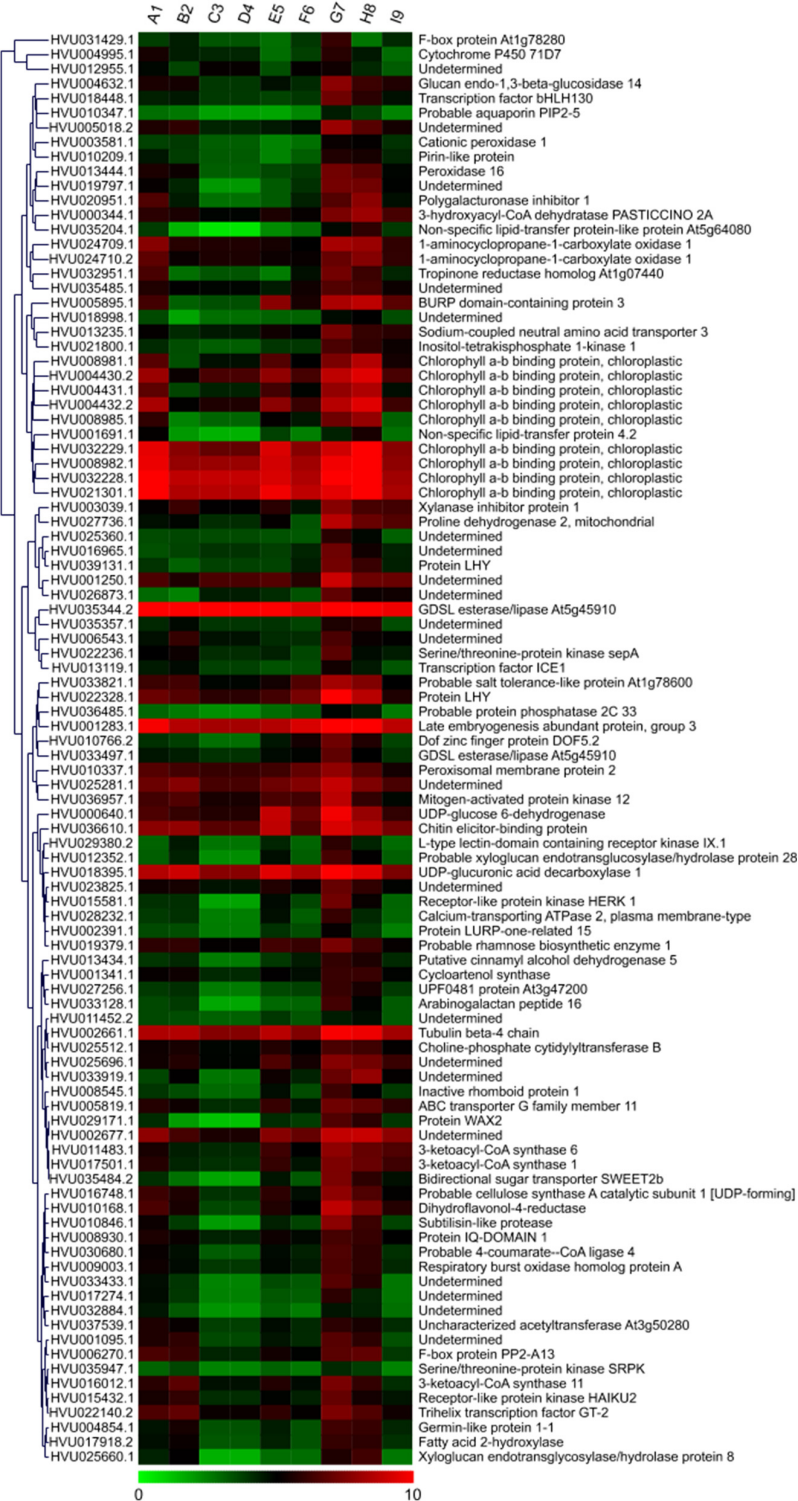
Gene expressions for the cluster IX showing down-regulated pattern under drought stress. The columns show the nine samples A1, B2, C3, D4, E5, F6, G7, H8, I9, while the rows show the log-transformed RPKM values of 98 genes in cluster IX, respectively. Hierarchical clustering of expression pattern for genes in was shown at the left of heat-map figure

The expression patterns and cluster analysis were conducted by *Mev* v4.7.4 software with K-Means clustering method and Pearson correlation as distance calculation method. The number of clusters is set to nine. Gene expression pattern for 853 potential drought-resistant related genes clustering into nine clusters (Fig. 2). 93 Genes in cluster IV were obviously down-regulated in low RSMC level, while 108 genes in cluster VI and 119 genes in cluster VIII were both up-regulated. Interestingly, 98 genes in cluster IX were up-regulated in the early phase of recovery process from sample G7 to H8 (Fig. 3). Furthermore, cluster IX shows up-regulation profile both in high and low RSMC level which indicates stress response to abnormal water content in soil. The other genes expression for the cluster IV, VI, VII, and VIII showed down-regulated pattern under drought stress (Additional file 1: Figure S1).

### GO and KEGG analyze DEGs

**Fig. 4:**
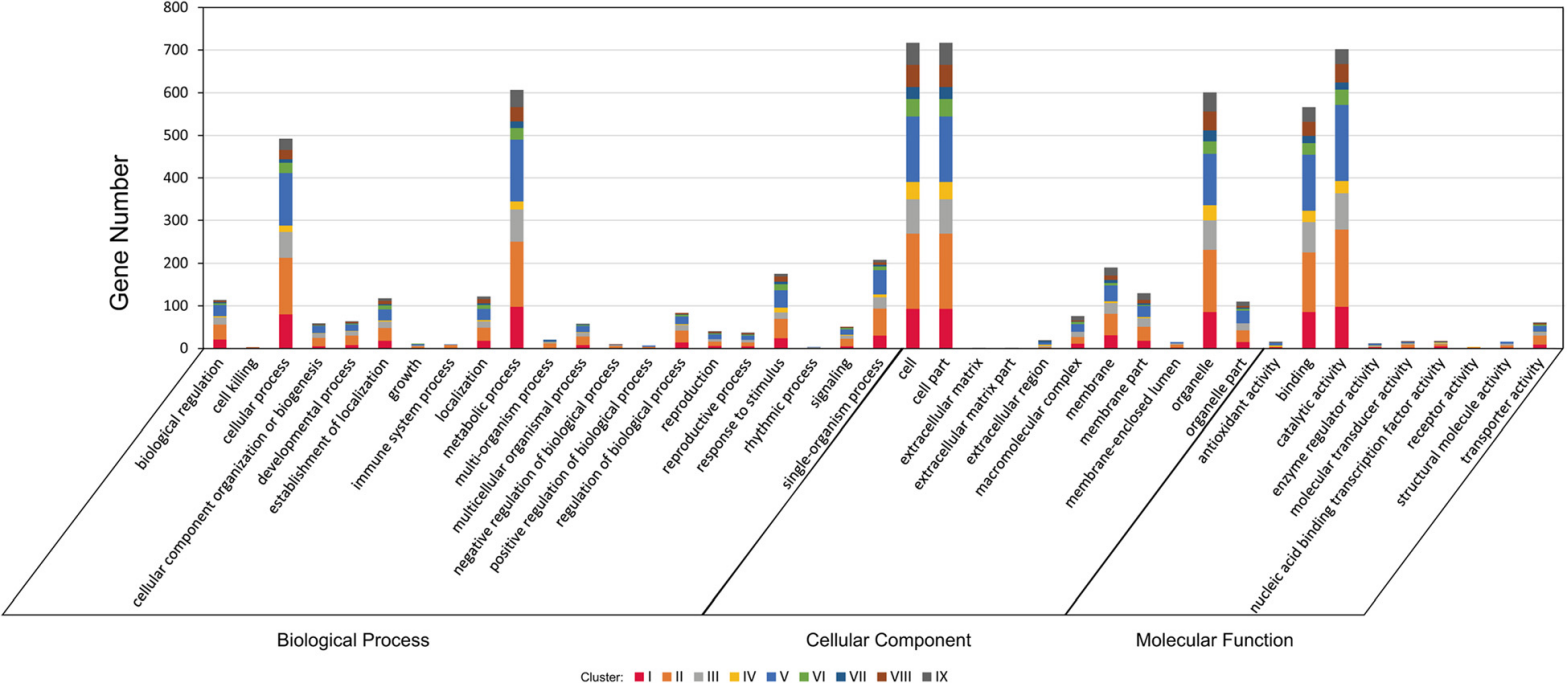
GO annotation of these 853 genes in each category. X-axis indicates GO terms, while the Y-axis indicates the percent of DGEs

**Fig. 5:**
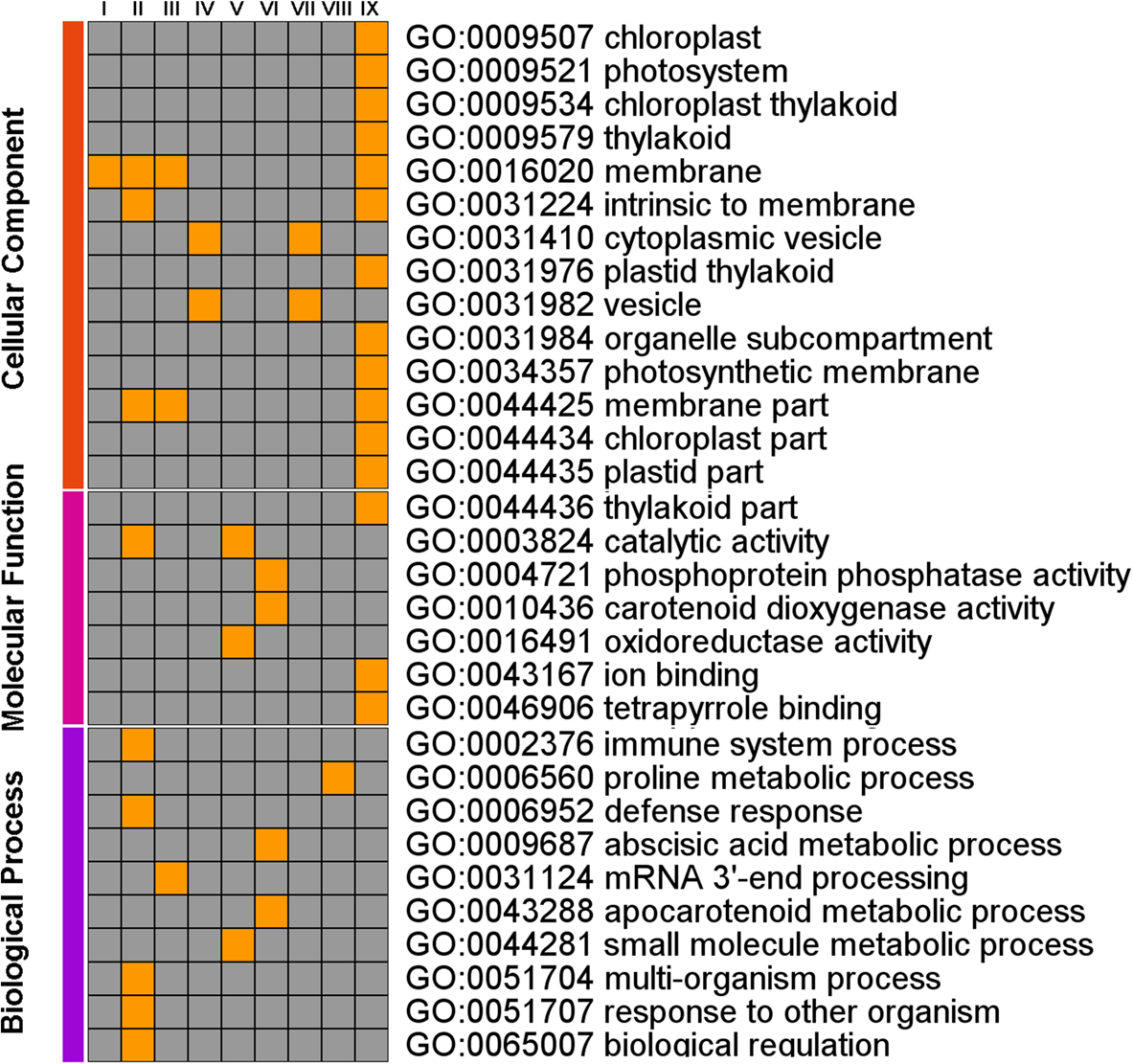
GO terms distribution in nine clusters (I ~ IX). Each column indicated each cluster, and each row indicated GO degree of enrichment. Red indicated greater enrichment, whereas the grey indicated lower enrichment

The combination of above two strategies results in a final 853 DEGs. We then conducted GO annotation and KEGG pathway analysis of these 853 genes. The assigned functions of these genes covered a broad range of GO categories (Fig. 4). Under the cellular component category, the parts of membrane-bounded organelle, cytoplasmic membrane-bounded vesicle, intrinsic to membrane, cytoplasmic part, intracellular part, plastid part, chloroplast part were prominently represented. Under the category of molecular function, the parts of catalytic activity, transferase activity, transferring phosphorus-containing groups, ion binding, cation binding, oxidoreductase activity represented the majorities of the category. For the biological process category, many genes were classified into the oxidation-reduction process, protein phosphorylation, phosphorus metabolic process, phosphate-containing compound metabolic process, regulation of biological process, biological regulation, transport, and cellular ketone metabolic process. GO enrichment of nine clusters were illustrated in Fig. 5. Of the listed twenty-one GO terms, majority GO terms fell into cluster IX. Tetrapyrrole binding, photosystem and photosynthetic membrane were the most affected in cluster IX. Only one GO term was included in cluster I and cluster VIII, respectively. GO terms focused on cellular component category, whereas DEGs were almost balanced distribution in three categories.

KEGG pathway analysis showed that these genes were mainly located in RNA transport, mRNA surveillance pathway, plant hormone signal transduction, defense-related gene induction, and glycerophospholipid metabolism pathway (Table 2).

**Table 2:** KEGG pathway of the 853 potential drought resistance related genes

Pathway	Gene Number	Pathway ID
Metabolic pathways	218	ko01100
RNA transport	134	ko03013
Biosynthesis of secondary metabolites	118	ko01110
mRNA surveillance pathway	109	ko03015
Plant hormone signal transduction	56	ko04075
Plant-pathogen interaction	53	ko04626
Glycerophospholipid metabolism	40	ko00564
Endocytosis	37	ko04144
Ether lipid metabolism	35	ko00565
Starch and sucrose metabolism	26	ko00500
Phenylpropanoid biosynthesis	24	ko00940
Cysteine and methionine metabolism	23	ko00270
Pyrimidine metabolism	23	ko00240
Purine metabolism	20	ko00230
Galactose metabolism	20	ko00052
Spliceosome	19	ko03040
RNA polymerase	17	ko03020
Circadian rhythm – plant	17	ko04712
Flavonoid biosynthesis	15	ko00941
Pentose and glucuronate interconversions	15	ko00040
Amino sugar and nucleotide sugar metabolism	14	ko00520
Stilbenoid, diarylheptanoid and gingerol biosynthesis	14	ko00945
Fructose and mannose metabolism	14	ko00051
Glycolysis/Gluconeogenesis	13	ko00010
Photosynthesis - antenna proteins	13	ko00196
Phenylalanine metabolism	12	ko00360
Protein processing in endoplasmic reticulum	11	ko04141
Carotenoid biosynthesis	11	ko00906
Limonene and pinene degradation	10	ko00903
Cyanoamino acid metabolism	10	ko00460

### Drought defense-related genes and pathways

**Fig. 6:**
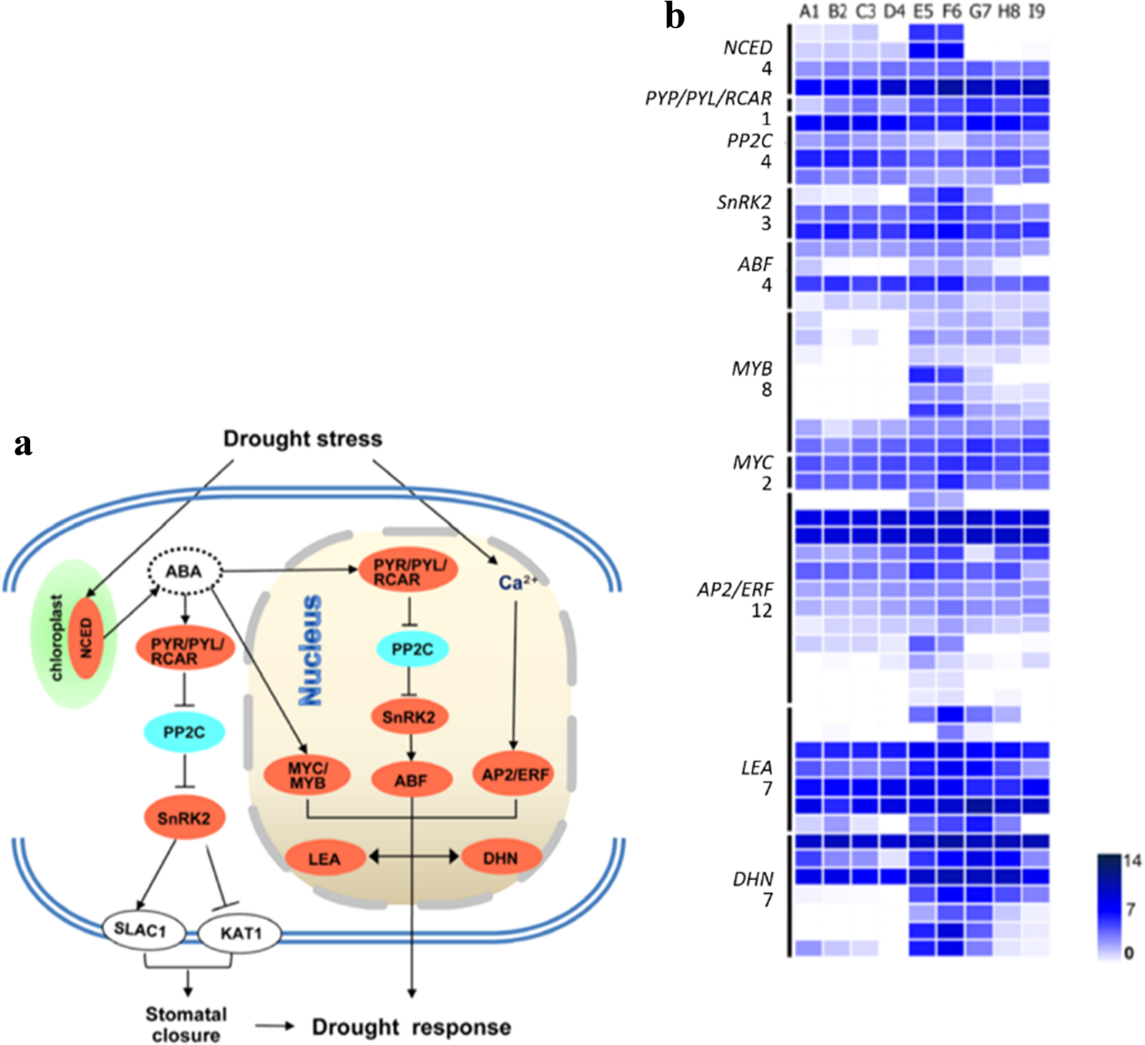
Expression of stress adaptation related genes. **a**, The typical ABA-dependent and independent signaling pathways responsive to drought stress. Red and blue ellipses indicate up-regulated and down-regulated genes identified by transcriptomes under drought stress, respectively. **a** was described by Mustilli et al. [22] and Yoshida et al. [23]. **b**, The expression pattern of representative genes allocated in above mentioned pathways. The expression levels were assessed by log2-transformed RPKM values. The A1-F6 indicates the relative soil moisture content (RSMC) of 33.4, 27.5, 21.1, 15.5, 9.8 and 4.8 %, respectively, and G7-I9 indicates the 2 h, 4 h and 8 h after water recovery from 4.8 % to 33.4 %, respectively. *NCED*, 9-cis-epoxycarotenoid dioxygenase; *SnRK2*, SNF1-related protein kinase 2; *ABF*, *AREB/ABF*, *MYB/MYC*, *AP2/ERF,* transcription factors, *LEA*, late embryogenesis abundant protein; *DHN*, dehydrin. The number below each gene indicates the member of each gene family

We also analyzed the genes related to ABA-dependent and independent signaling pathway of drought stress responses, including *NCED*, *PYR/PYL/RCAR*, *SnRK2*, *ABF*, *MYB/MYC*, and *AP2/ERF* transcription factors. The typical ABA-dependent and independent signaling pathways responsive to drought stress was illustrated in Fig. 6a. The pathways were described by Mustilli et al. [25] and Yoshida et al. [26]. Almost all genes were up-regulated except *PP2C* down-regulated. Dehydration first induced the expression of *NCED* in chloroplast and Ca^2+^ accumulation in nucleus. ABA biosynthesis induction of *NCED* triggered *PYR/PYL/RCAR* and *MYB/MYC. PYR/PYL/RCAR* located both in nucleus and the outside blocked the expression of *SnRK2* by inhibiting *PP2C* synthesis. *SnRK2* located in cytoplasm may accelerate the expression of *SLAC1* but repress the expression of *KAT1* located in cell wall, and then result in stomatal closure. This was one response formation in cytoplasm to drought. *AP2/ERF* caused by Ca^2+^ accumulation, *ABF* acted by *SnRK2*, and *MYB/MYC* induced by ABA together promoted the expression of *LEA* and *DHN* in nucleus, and finally mediated drought response. Figure 6b exhibited the expression pattern of representative genes allocated in Fig. 6a mentioned pathways. The expression of *NCED* dramatically decreased during water recovery. *PYR/PYL/RCAR*, *PP2C*, and *MYC* still kept a relative balance expression. The expression of *MYB*, *LEA*, *DHN* rapidly increased when RSMC reached 9.8 %. All genes presented an abundant expression during the RSMC maintained from 9.8 % to 4.8 %.

## Discussion

Drought, salinity and cold are the major environmental factors impacting on survival and productivity of hulless barley in Tibetan Plateau of China. Barley is known to be relatively tolerant to abiotic stresses among the major cereal crops and, thus, is often grown in more marginal sites [27]. Tibetan hulless barley cultivar, Himalaya 10, has developed a strong tolerance and adaptation to drought deficit. Using high-throughput RNA sequencing technology, we compared in detail the transcriptional differences and overlap between different levels of RSMC and water recovery, and display the changes of gene expression in Tibetan hulless barley during the whole drought response process.

In this study, the mRNA of Himalaya 10 with good drought tolerance was sequenced using Illumina HiSeq™ 2000 in the manner of PE91, with Sera-mag Magnetic Oligo (dT) Beads. A clear bioinformatic map of mRNA involved in multiple biological processes was produced. As a result, 53.99 G clean reads were collected from nine samples under different RSMC and water recovery, which met the requirements for further analysis. Saturability analysis indicated a qualified coverage of most genes based on our data size. In addition, the clean reads of Q20 occupied over than 95 % of the total, suggesting high quality sequencing. TopHat package was used to blast the transripome data to the Tibetan hulless barley. It has been found that 71.6 % of the reads were mapped to the reference genome. Major mapping reads indicated reliable transcriptome data. These non-mapped tags most likely represent regions where the reference genome is incomplete [28], or there are allelic sequence differences between the reference genome and the cultivar Himalaya 10 used in this study. Another reason may be that RNA-seq data for reference genome annotation should represent all major tissue types, developmental stages and responses to abiotic and biotic stresses [29].

DGE global analysis provided a comprehensive dataset responding to drought stress in leaves of hulless barley seedlings. We identified nine clusters for all DGEs and coarsely assigned them to 31 functional categories (*p* <0.05) (Fig. 4). Interestingly, there were no other overlapping GO functional enrichments between clusters except catalytic activity, membrane, vesicle, indicating that these genes of different clusters were predicted to be involved in many plant biological processes, including defense [30]. These GO functions were enriched in IX cluster (Fig. 5). Tetrapyrrole binding, photosystem and photosynthetic membrane were the most affected in IX cluster. The former finding implied that tetrapyrroles were the structural backbone of chlorophyll and heme were essential for primary photochemistry, light harvesting, and electron transport [31]. Tetrapyrrole-binding proteins of cHBP1 and cHBP2 have properties suitable for tetrapyrrole carrier proteins [32]. Tetrapyrrole binding protein of genomes uncoupled 4 (GUN4) regulates chlorophyll synthesis and plastid-to-nucleus signal transduction by binding both the product and the substrate of Mg-chelatase, an enzyme that produces magnesium-protoporphyrin IX (Mg-Proto) [33]. Tetrapyrrole biosynthesis had recently been implicated in wilting avoidance [34, 35]. Heme mediated chloroplast-to-nucleus signalling upon drought stress [36, 37]. Enhanced tetrapyrrole biosynthesis was likely to confer drought tolerance via retrograde signaling and induction of drought-responsive gene expression [38].

Pathway enrichment analysis revealed 30 pathways were significantly affected by drought stress (Table 2). Plant hormone signal transduction pathway, for instance, ABA signal transduction pathway was a significant different during the whole drought response process in our study. Transcription factors of *NCED*, *PYR/PYL/RCAR*, *PP2C*, *SnRK2* located in cytoplasm together lead to stomatal response and then transcription factors of *PYR/PYL/RCAR*, *PP2C*, *SnRK2*, *ABF*, *MYB/MYC*, *AP2/ERF*, *LEA*, and *DHN* located in nucleus, together mediated drought response (Fig. 6a). Soil drying first inducted ABA release in roots and then was distributed throughout the plant via the transpiration stream [18]. ABA induced reduction of leaf growth rate and stomatal closure, which triggered stress proteins and various metabolites to protect cells against drought stress [4, 37] when ABA was produced endogenously via water deficit, and then plant tolerance to drought was increased [39–41]. Drought stress signals can also be generated by osmotic stress-induced Ca^2+^ expansion, which promoted Ca^2+^ channels and induces protein kinases and resulted in drought-responsive gene expression [42–44]. Du et al. [45] has compared *DHN* between wild barley and Tibetan hulless barley associated with drought stress resistance. Liang et al. [46] indicated that *LEA* genes (*HVA1* and *Dhn6*) might participate in adaptive responses to water deficit in different ways in Tibetan hulless barley. Qian et al. [47] indicate that the differential *HVA1* gene has a functional role in the dehydration tolerance in Tibetan hulless barley.

## Conclusions

In summary, this study provided a comprehensive analysis of drought-responsive genes and transcriptome expression profiles of Tibetan hulless barley leaves by combined DGE, RNA-seq, and computational approaches. Our results revealed 853 potentially drought-responsive genes in Tibetan hulless barley. We analyzed the genes related to ABA-dependent and independent signaling pathway of drought stress responses. Additionally, we also observed the genes related to tetrapyrrole binding of drought stress responses. This result filled up drought-resistant related genes in Tibetan hulless barley in the available literature. Characterizing the components of these pathways will contribute to improve drought tolerance in Tibetan hulless barley.

## Methods

### Plant growth under drought condition

**Fig. 7:**
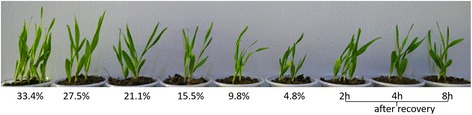
Himalaya 10 underwent a series of RSMCs (33.4, 27.5, 21.1, 15.5, 9.8 and 4.8 %) and rewatered conditions to 33.4 % for 2 h, 4 h and 8 h

An elite hulless barley cultivar Himalaya 10, with good drought tolerance, is used for drought tolerant gene analysis. The RSMC of the original soil sample using for planting were measured and adjusted to 33.4 % by adding proper volume of water. The seedlings of Himalaya 10 were growing under same condition in a greenhouse with a temperature of 23 °C/15 °C (day/night) and a relative humidity of 10–20 %. Three plants from each pot at given condition were considered as biological replicates. Prior to drought stress treatment, these seedlings were well-watered by supplying with proper amount of water every two days to maintain the RSMC at 33.4 %. Different RSMCs at stable status were described by Zhang et al. [48] and Wang et al. [49]. Based on their descriptions, we weighted each pot twice at 8:00 a.m. and 8:00 p.m. Since the soil and plant of each pot was relatively constant, we only add certain water to maintain the RSMC of each pot. Irrigation was removed when the seedlings grew to two-and-a-half leaf stage (18 days after sowing) at six different levels by deficit altering supply water. Eight days after drought stress, leaf samples were harvested from all six groups. The drought stress levels of these groups were evaluated with their score of RSMC (33.4, 27.5, 21.1, 15.5, 9.8 and 4.8 %). After drought treatment, the remaining seedlings of 4.8 % RSMC group were rewatered to restore the RSMC to 33.4 %, leaf samples during rehydration (2 h, 4 h and 8 h after RSMC recovered to 33.4 %) were also collected (Fig. 7). All these leaf samples mentioned above were fast frozen in nitrogen immediately and stored at −80 °C for RNA-seq analysis respectively.

### RNA isolation and construction of hulless barley RNA-seq library

After the total RNA extraction and DNase I treatment, we used magnetic beads with Oligo (dT) to isolate mRNA. The mRNA molecules were fragmented into 200 bp and cDNAs were synthesized taking mRNA fragments as templates. The cDNA fragments were purified and resolved with EB buffer for end repair, single nucleotide A (adenine) addition and connection of adapters. After PCR amplification, Agilent 2100 Bioanaylzer and ABI StepOnePlus Real-Time PCR System were used in quantification and qualification of the sample library. At last, the 200 bp library was sequenced using Illumina HiSeq™ 2000 in the manner of PE91. The clean reads were obtained by filtering out adaptor-only reads, reads containing more than 5 % unknown nucleotides, and low-quality reads which the percentage of low quality bases (base quality ≤ 10) is more than 20 %.

### Gene expression levels and identification of differentially expressed genes (DEG)

The gene expression was calculated by RPKM method (reads per kb per million reads) [50]. The RPKM method is able to eliminate the influence of different gene length and sequencing discrepancy on the calculation of gene expression, which could facilitate comparing the difference of gene expression among samples.

DEGs were identified between two samples in the method described below, which was intend to detect the significance of digital gene expression profiles [51]. The clean reads for gene A is x, x yields to the Poisson distribution. The total clean read numbers of the sample 1 and sample 2 are N1 and N2 respectively. Gene A holds x and y reads in sample1 and sample2. The probability of gene A expressed equally between two samples could be calculated by the following formula:

$$ 2{\displaystyle \sum_{i=0}^yp}\left(i/x\right)\kern1em \left( if{\displaystyle \sum_{i=0}^yp\left(i/x\right)\le 0.5}\right) $$

Also, the p value was corrected for false positive (type I errors) and false negative (type II errors) using FDR method [52]. We use FDR ≤ 0.001 and the absolute value of Log2Ratio ≥ 1 as the threshold to identify DEGs.

Gene Ontology (GO), pathway annotation and enrichment analyses were based on the Gene Ontology Database (http://www.geneontology.org/) [53] and Kyoto Encyclopedia of Genes and Genomes (KEGG) pathway (http://www.genome.jp/kegg/) [54], respectively. When we investigated pathways in which different genes were involved and enriched, q-value was used to aid identification according to the previous description [55].

### DEGs and drought defense-related genes

Samples A1, D2, C3, D4, E5 and F6 were divided into two groups: one includes A1, B2 and C3, which is under water-sufficient conditions; the other consists of D4, E5 and F6, which is under withholding water conditions. Then, the comparison analysis between the two groups was performed use Noiseq method [56], and these DEGs were finally obtained. The pairwise comparison was also executed among samples F6, G7, H8 and I9. We filtered genes whose RPKM value are below five in all four samples and removed genes that the frequency of occurrence is less than three in pairwise comparison, and then we got those DEGs. The combination of above two strategies results in a final DEGs. We then conducted GO annotation and KEGG pathway analysis of these genes. The expression patterns and cluster analysis were conducted by *Mev* v4.7.4 software [57] with K-Means clustering method and Pearson correlation as distance calculation method.

### qPCR analysis

To validate the results of the RNA-seq data, expression of the genes the same samples used for Transcriptome-seq analysis was performed by real-time quantitative reverse transcription-polymerase chain reaction (RT-PCR) with the fluorescent intercalating dye SYBRGreen in a detection system (Opticon 2; MJ Research, Waltham, USA), using a hulless barley gene (*HvADP*) as a standard control [58]. A two-step RT-PCR procedure was performed in the experiments. First, 2 μg of purified total RNA was reversely transcribed into cDNAs which were used as templates for PCR reactions using gene-specific primers (Table 3). Second, quantitative PCR was performed using PCR Master Mix (Toyobo, Osaka, Japan) according to the manufacturer’s instructions. Relative quantification of gene expression was determined using the comparative Ct method. To achieve optimal amplification, PCR conditions for each primer combination were optimized for annealing temperature, and PCR products were verified by melting curve analysis and confirmed on an agarose gel. Mean values and standard errors were calculated from three independent experiments with three biological replicates of hulless materials, and the data were normalized with the relative efficiency of each primer pair.

**Table 3:** Primers of qRT-PCR assay used for eight RNA-seq libraries in this study

Gene_id	Amplicon size (bp)^a^	Forward primer (5′- > 3′)	Reverse primer (5′- > 3′)
HVU014442.1	142	ATTTCTTCGACTGGGGCCTG	ATGACCTTGCCGTCGATCTC
HVU014441.1	190	CAGGAGCCTGAGTAGATGCG	AATGAGAGGCCGACCACAAG
HVU029679.1	194	AAGAAACTGATCCGAGGCGG	ATTCGTCCGGCCCGTATTTT
HVU021835.1	180	GCGGCTATATCCCACCTTCC	AAGAGCTGAGGTGAAGCGAC
HVU015180.1	208	TGGAGAAACGGATCGAAGCC	TGCAGCCCAGCTAGAAAAGG
HVU021802.1	206	CCTTCACCTCCAGGAACGTC	CACTCCGATTCCACTTGGCT
HVU025938.1	180	TGTCACCGCTGAACCAATCA	TTCACTGGGATTCACGGACG
HVU026759.1	86	GAAGTCGCGCGAATCTGTTC	GAGCAGAGCAGCCAGATCAA
HVU015112.1	215	AAGATCCCAACAAGGCGAGG	CCTGGCTTGCTCCTCTTTGA
HVU001215.1	229	AAGCATGCCGTCTTACACCA	GGCCATCGGAGAGTGCATTA
HVU012352.1	163	TGGCGTGTCAGCTGAGATTT	GGCCGTGAAGGACCAAAAAC
HVU033128.1	214	CCATCAGACTGTGGCGTCTT	GAACCCTCTGCGCATAGACA
HVU023469.1	244	AGCACTACTACGGCACCAAC	CTGAAACCGGCGTACTCCTT
HVU007307.1	216	AGATGGGGTGCGAACTTGAG	CAGGGACAGGACCATCCAAC
HVU011039.1	115	GGTGATCATGCGTGTGTTCG	CTTCTTGGCCGAGTCCTTGT
HVU033209.1	234	AGCCCAAACCTACCAAGCTC	ATCATAGCGCAGGAGCCATC
HVU000918.1	87	GTAGCTATCCACGGTCACGG	TGGAACTGTAAGCGTCCACC
HVU007765.1	208	CGCATGCTGATGGAAAAGGG	ATTGCACCGCACTCAACAAC
HVU006724.1	135	TTTGTGTGGGGAGGTCGATG	CATGCACTCTTCGGTGACCT
HVU016310.1	245	TGAGGATGAAGCGAGTGCAG	TGGGGACTAGCACGCAAAAT

^a^The amplicon lengths for all the genes are within 70–250 bp range to satisfy the requirement for the primer pairs used for real time RT-PCR analysis using SYBR green according to manufacturer’s guide

### Declarations

#### Ethics approval and consent to participate

Not applicable.

### Consent for publication

Not applicable.

### Availability of supporting data

The transcriptome raw data of Tibetan hulless barley under drought stress have been deposited in the NCBI under the accession number PRJNA316037 (http://www.ncbi.nlm.nih.gov/bioproject/PRJNA316037).

## Additional file

Additional file 1: Figure S1. Gene expression for the cluster, IV (a), VI (b), VII (c), and VIII (d) showing down-regulated pattern under drought stress. The columns show the 9 samples A1, B2, C3, D4, E5, F6, G7, H8, I9, while the rows show the log-transformed RPKM values of 93 genes in cluster IV, l08 genes in cluster VI, 52 genes in cluster VII, and 119 genes in cluster VIII, respectively. Hierarchical clustering of expression pattern for genes in was shown at the left of heat-map figure. (DOCX 950 kb)

## Abbreviations

ABA: abscisic acid
ABF: ABRE binding factor
ABRE: ABA-responsive element
AP2/ERF: APETALA2/ethylene-responsive factor
AREB: ABA-responsive element binding factor
DEGs: differentially expressed genes
DHN: dehydrin
LEA: late embryogenesis abundant protein
NCED: 9-cis-epoxycarotenoid dioxygenase
PP2C: type 2C protein phosphatases
PYR/RCAR: pyrabactin resistance/regulatory component of ABA receptor
qRT-PCR: quantative real-time PCR
ROS: reactive oxygen species
RSMC: relative soil moisture content
RT-PCR: reverse transcriptase polymerase chain reaction.
SnRK2: sucrose non-fermenting 1-related protein kinases2
