# Supplementary material for: Transcriptome analysis revealed the drought-responsive genes in Tibetan hulless barley
Source: BMC Genomics. 2016 May 20;17:386. doi: 10.1186/s12864-016-2685-3 (PMC4875595; doi:10.1186/s12864-016-2685-3)
Supplement: Additional file 1: Figure S1. — Gene expression for the cluster, IV (a), VI (b), VII (c), and VIII (d) showing down-regulated pattern under drought stress. The columns show the 9 samples A1, B2, C3, D4, E5, F6, G7, H8, I9, while the rows show the log-transformed RPKM values of 93 genes in cluster IV, l08 genes in cluster VI, 52 genes in cluster VII, and 119 genes in cluster VIII, respectively. Hierarchical clustering of expression pattern for genes in was shown at the left of heat-map figure. (DOCX 950 kb) [file 12864_2016_2685_MOESM1_ESM.docx]

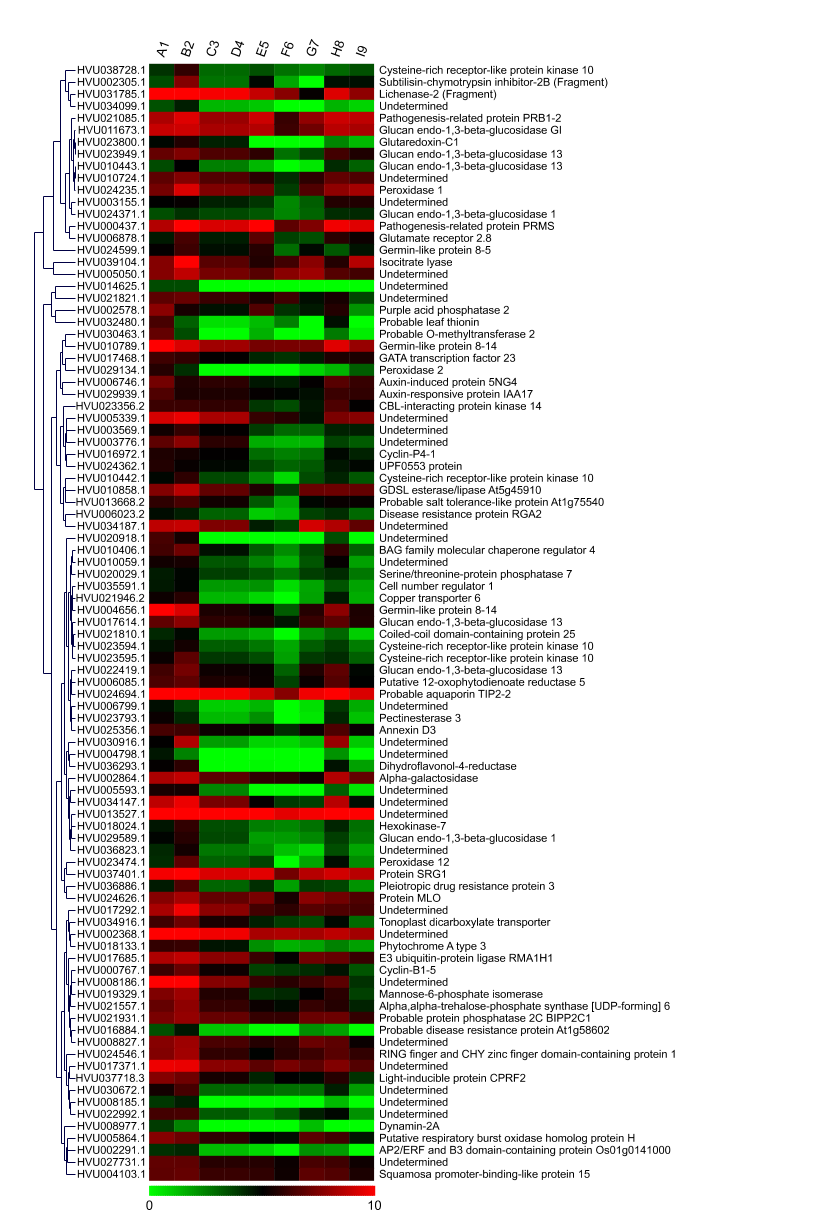

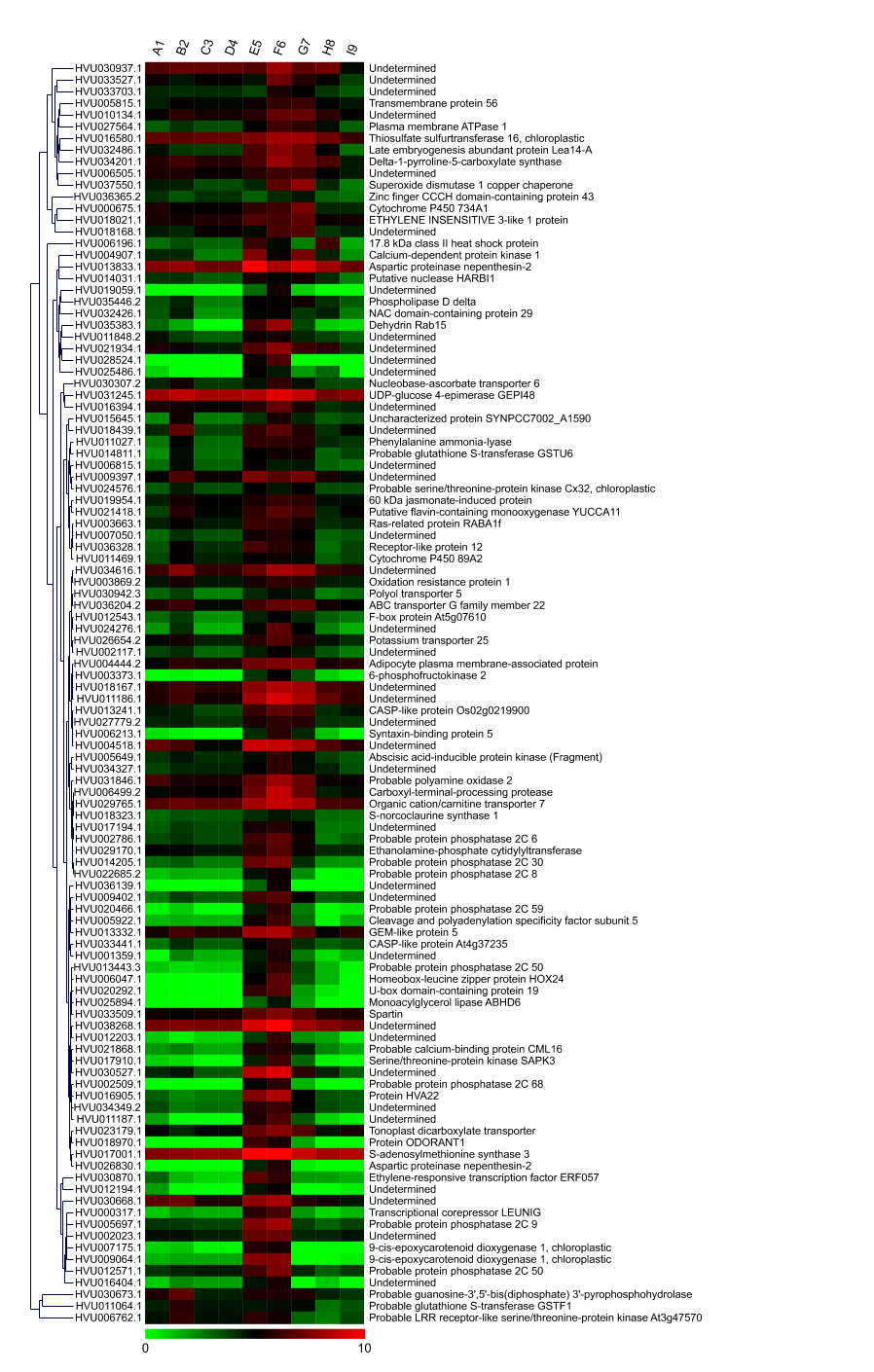

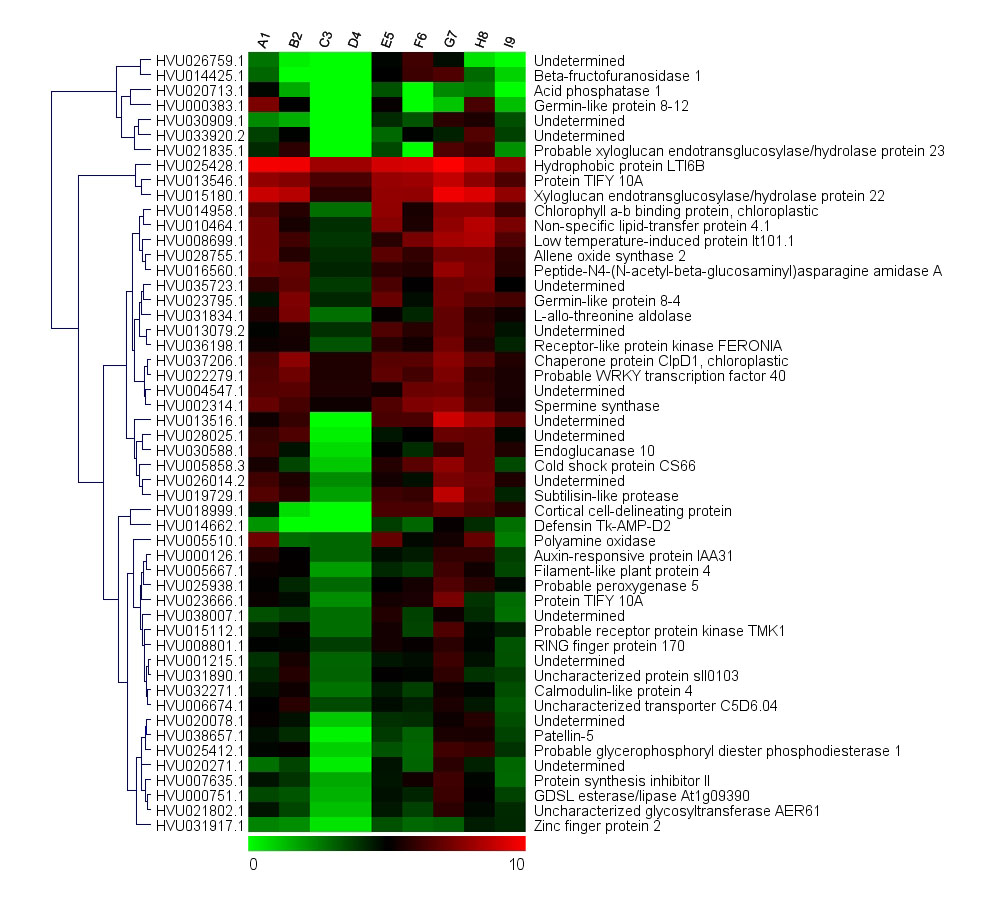


**Supplementary Figure 1a**

**Supplementary Figure 1b**

**Supplementary Figure 1c**


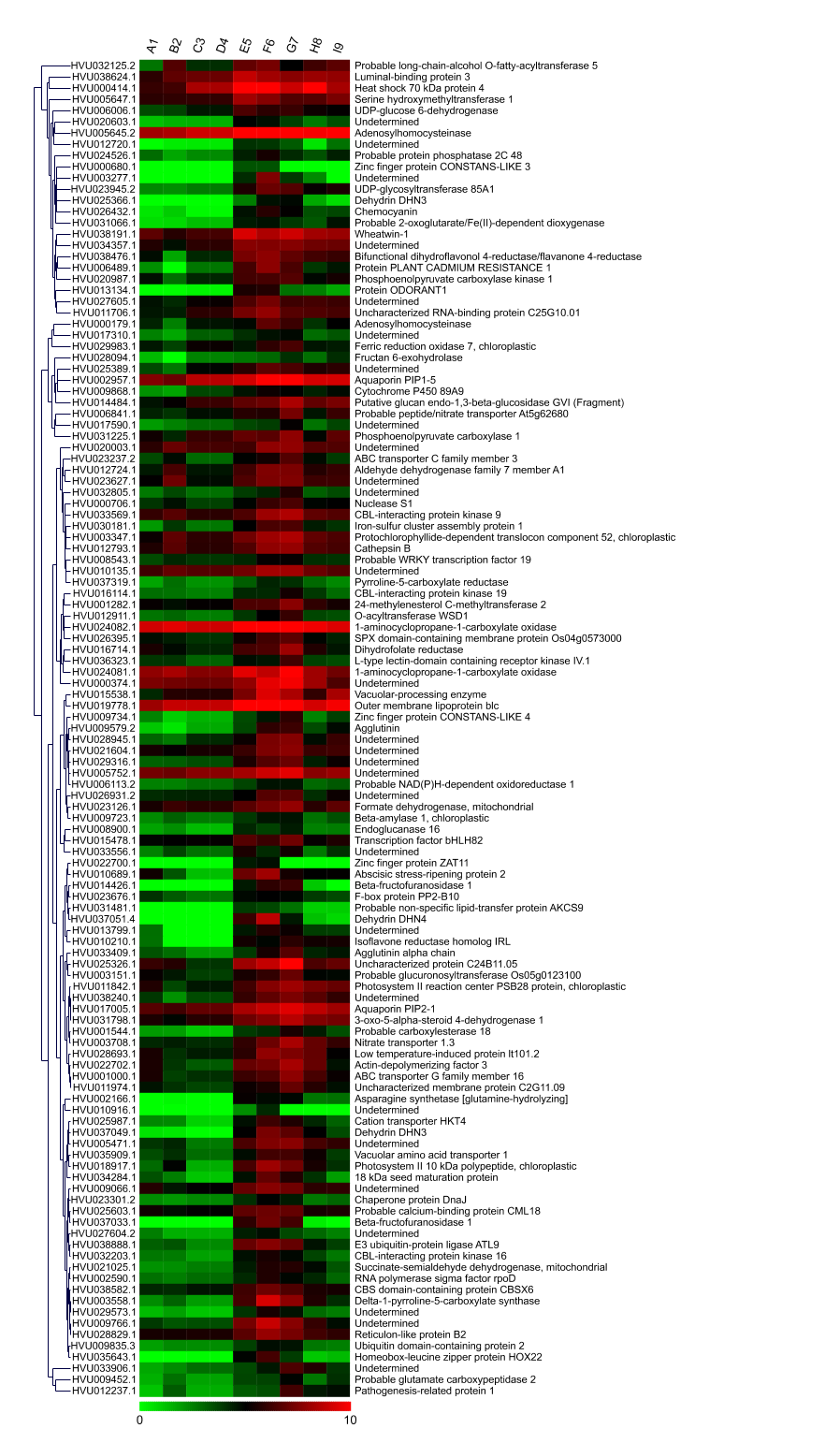


**Supplementary Figure 1d**

**Additional file 1: Figure S1.** Gene expression for the cluster, IV (a), VI (b), VII (c), and VIII (d) showing down-regulated pattern under drought stress. The columns show the 9 samples A1, B2, C3, D4, E5, F6, G7, H8, I9, while the rows show the log-transformed RPKM values of 93 genes in cluster IV, l08 genes in cluster VI, 52 genes in cluster VII, and 119 genes in cluster VIII, respectively. Hierarchical clustering of expression pattern for genes in was shown at the left of heat-map figure.
